# High acceptability and viral suppression of patients on Dolutegravir-based first-line regimens in pilot sites in Uganda: A mixed-methods prospective cohort study

**DOI:** 10.1371/journal.pone.0232419

**Published:** 2020-05-27

**Authors:** Vennie Mbaziira Nabitaka, Pamela Nawaggi, Jennifer Campbell, James Conroy, Joseph Harwell, Kinanga Magambo, Caroline Middlecote, Benvy Caldwell, Cordelia Katureebe, Norah Namuwenge, Rita Atugonza, Andrew Musoke, Joshua Musinguzi

**Affiliations:** 1 Clinton Health Access Initiative, Kampala, Uganda; 2 Ministry of Health, Kampala, Uganda; University of Ghana College of Health Sciences, GHANA

## Abstract

Uganda adopted the integrase inhibitor dolutegravir (DTG) as part its preferred first-line HIV treatment regimen in 2018. Prior to the national rollout, the Uganda Ministry of Health and Clinton Health Access Initiative (CHAI) launched a pilot study in July 2017 aimed at better understanding patients’ and prescribers’ experience and acceptability of DTG. Patients were enrolled in the study if they were newly initiating treatment or switched from an NNRTI regimen due to intolerance. Patients were followed up for 6 months after initiation onto DTG and acceptability and experiences were assessed through questionnaires at one-month and six-month follow-up visits. In addition to acceptability side effects of patients on DTG regimens were assessed. Analysis was conducted using MS Excel and SAS 9.4 and confidence intervals were adjusted for facility level clustering. A total of 365 patients from 6 study sites were enrolled in the study, of whom 50% were treatment-experienced and 50% treatment naïve. 325 patients completed the 6 months of follow-up. Survey results showed a high level of acceptability (more than 90%) of DTG-containing regimens for both categories of patients during the from one-month and six-months interviews. The rate of self-reported side effects amongst patients was 33% overall and higher for experienced (37%) than naïve (29%) patients at 6 months. Although frequencies declined between month-1 and month-6, the changes were not statistically significant. Almost all patients (94%) were virally suppressed at 6 months. Overall, the study findings showed a very high level of acceptability of Dolutegravir-based regimens across both experienced and naïve patients. The overall viral suppression rate in this cohort was 94% at six months of taking DTG-based regimen.

## Introduction

Uganda adopted the integrase inhibitor dolutegravir (DTG) as its preferred first-line HIV treatment regimen in 2018. The national roll-out began in September 2018. Prior to the national rollout, the Uganda Ministry of Health and Clinton Health Access Initiative (CHAI) launched a 6-month follow-up pilot study in July 2017 aimed at better understanding patients’ and prescribers’ experience and acceptability of DTG.

### HIV treatment program in Uganda

Uganda has made commendable progress in accelerating of the availability of HIV care and treatment services since the program’s inception. Treatment guidelines that were adopted in 2016 expanded treatment eligibility to all adults and adolescents with the universal “Test and Treat” policy which was scaled-up nationally in 2017. In Uganda, about 96% of patients on ART are currently taking first-line regimens [1]. At the start of the study, the preferred first-line regimen was Tenofovir/ Lamivudine/ Efavirenz (TLE) which was being used by 45% of adult first-line patients [2].

Non-Nucleoside Reverse Transcriptase Inhibitor (NNRTI) based regimens have been preferred first-line regimens for adults in Uganda since the beginning of the ART program over two decades ago. The continued effectiveness of NNRTI-based regimens has been questioned as the documented levels of transmitted drug resistance to them have increased in Uganda and elsewhere [3, 4]. Studies have shown alarmingly high (9–12%) levels of transmitted resistance in Uganda which introduced ART well ahead of neighboring countries [5].

The proportion of patients receiving treatment other than first-line has increased from 2.9% in 2010 to 5.8% in 2018, which is cost prohibitive, at triple the cost of first-line [6]. In addition to achieving better treatment outcomes, introducing the new Integrase Strand Transfer Inhibitor (INSTI) class of ARVs can help to reduce migration to more expensive second and third line drugs and curb antiretroviral (ARV) drug resistance thus allowing the country to instead channel the savings towards initiating more patients on first-line ART, as is the goal under the “Test and Treat” policy [7].

### Dolutegravir as a first-line drug

In December 2015, the World Health Organization (WHO) released updated ART guidelines for HIV treatment and use of antiretroviral drugs that included use of more optimal drugs such as DTG as an alternative in first-line ART regimens. DTG is an INSTI that is currently available as a single pill of 50mg and in combination with Tenofovir and Lamivudine. The SINGLE study showed that DTG was non-inferior to efavirenz (EFV); virologic efficacy of 88% vs. 81% P = 0.003; a treatment difference of 7%, 95% CI = 2–12% at 48 weeks [8, 9]. SINGLE also reported fewer discontinuations due to adverse effects with DTG (2%) vs. EFV (10%). DTG patients also achieved faster time to viral suppression (median time to suppress = 28 vs. 84 days). Introduction of DTG as a first-line regimen therefore, not only has clinical benefits for the patients, but also public health benefits including promoting rapid viral suppression potentially reducing the number of new HIV infections.

### Rationale for the study

With the growing patient numbers on ART and prohibitive costs of second-line treatment, it is imperative to keep patients on a highly efficacious and optimal first-line regimen with few adverse effects in order to reduce the growing need of second-line treatment. For this reason, the Ministry of Health piloted the use of dual-pill Tenofovir/Lamuvudine and DTG singles (TL+D) as a preferred first-line treatment option in selected high volume sites in Uganda. This work was designed to reduce barriers to national roll-out by understanding the perspectives on using the drug from the patients’ as well as the clinical response in a subset of patients.

## Methods

This was a mixed-methods descriptive, observational prospective cohort study. The patient cohort included patients already on treatment that were experiencing intolerance to their NNRTI regimen (treatment-experienced patients) as well as newly initiating patients. Pregnant women or women intending to become pregnant during the study period were excluded. Patients with TB were not excluded.

Two sample sizes were calculated; one for treatment-naïve patients and another for patients switching to a DTG-based regimen using the standard proportion formula below was used for calculating sample size. The sample size estimate was then adjusted for a survey design with a conservative design effect of 2 that is used for cluster and survey sampling.

$$sample\;size=\frac{z{\left(1-\frac{\alpha }{2}\right)}^{2}p(1-p)}{d^{2}}\;where\;\alpha =0.05,z=1.95,d=2\;and\;p=expected\;proportion$$

For treatment-naïve patients, a sample size of 146 was calculated using an expected proportion (p) of 0.05, reflecting the expected proportion of patients that will report severe side effects to DTG [10]. For the arm of the ART-experienced patients switching to a DTG-based regimen from an NNRTI based regimen due to intolerance, a sample size of 174 was calculated using an expected proportion of 0.85, reflecting the expected proportion of patients that will prefer the DTG-based regimen to their previous regimen [11–13]. Patients were enrolled over a three-month period from end of July 2017 through October 2017 and followed up for 6 months.

There were 6 study sites all located in central Uganda. The sites were purposively selected to represent a mix of facility levels from the health center 3 (lowest) and the referral hospital level (highest). High volume facilities (at least 1,000 patients enrolled in chronic HIV care) were considered for inclusion while excluding faith-based, military or for-profit private clinics in an effort to best represent nationwide public clinic programs.

The primary objective was to describe the experience and acceptability of using DTG as part of a first-line regimen from the ART prescribers and patient’s perspective. This paper focuses on the patients’ perspective. The secondary objective of the study was to assess the clinical experience of the patients as documented in their clinical records by their health care providers.

Patient acceptability assessments were conducted through one-on-one interviews between the patient and health counselor using questions on paper based scripts in English or a local dialect (Luganda) depending on the patient’s preference. Patients were assessed through 3 questions: all treatment-experienced patients were asked if they preferred their current regimen to the previous one (question one) and if they believed DTG is easier to take than their previous regimen (question two). Additionally, all patients, both experienced and naïve were asked if they would recommend the drug to other patients (question 3). At the end of the interview, respondents were also asked an open ended question to describe the new ARV medication in order to validate prior responses.

In addition to acceptability, side effects of DTG based regimens were assessed through two questions in the patient interviews. All patients were asked if they had experienced any of the 19 pre-listed side effects, and if so, to rank the severity of the side effect on a 5-point Likert scale with 5 being the most severe. Additionally, treatment-experienced patients were asked whether any pre-existing side effect, of the 18- side effects listed, had improved, resolved, remained unchanged, or worsened since switching to DTG. The qualitative responses were analyzed for themes. At every patient visit, health care providers filled a patient record form indicating any opportunistic infections, adherence scores, side effects and all medications that the patient was taking (ART and others). At the 6-months’ visit, blood for a viral load test was taken and sent for analysis in the routine laboratory. The last health record was recorded at the time the 6-months’ viral load results of the patients were returned.

Trained data entrants entered all completed questionnaires and health record forms into Survey CTO (from hard copies) during the third month of the study and at the end of the study. Analysis was conducted in MS Excel and SAS 9.4. To test for differences amongst the lost-to-follow-up (LTFU) patients, chi-square tests for the proportions and a Mann-Whitney test was used for age (while adjusting for facility clustering except for when testing for differences in the facilities). To test for significance of changes in side effects reporting between month-1 and month 6, P-values were calculated using chi-square testing adjusting for facility level clusters.

Ethics approval for this study was obtained from Chesapeake/ Advarra (REF NO: Pro00022105), Makerere University School of Health Sciences Research and Ethics Committee (REF NO: 2017–045) and Uganda National Council of Science and Technology (REF NO: HS 2248). Written informed consent was sought from all patients prior to participation in the study.

## Results

### Baseline characteristics of participants

There were 365 patients enrolled in the study, of whom 50% were treatment-experienced and 50% treatment-naïve. There were more women (53%) than men (47%) and the median age was 35 years (Table 1). For the ART experienced group, the median time on ART prior to switching was 18 months (ranged from 2 weeks to 132 months).

**Table 1:** Baseline summary statistics of all patients that were enrolled in the study.

	Total	Tx Experienced		Tx Naive	
		n	%	n	%
**Total**	365	184	50%	181	50%
**Age (median)**	36 IQR = 16	37 IQR = 15		35 IQR = 14	
**Sex**					
Male	167	78	47%	89	53%
Female	198	106	54%	92	46%
**Facilities**					
Health Facility 1	70	42	60%	28	40%
Health Facility 2	73	45	62%	28	38%
Health Facility 3	71	34	48%	37	52%
Health Facility 4	60	32	53%	28	47%
Health Facility 5	69	35	51%	34	49%
Health Facility 6	22	10	45%	12	55%

Forty participants did not complete the study. Five participants discontinued due to pregnancy. The precautionary note on DTG use in women released by the World Health Organization came at the end of the study so the discontinuations after pregnancy diagnosis were based on the study protocol. 70% (p value <0.01) of patients who discontinued were ART naïve. Differences in age, sex and health facility for patients who discontinued were not significant. Table 2 below shows the reasons for discontinuation.

**Table 2:** List of reasons for discontinuation from the study.

Cause	No. of enrollees	Comment
Pregnancy	5	Overall 5 pregnancies were reported during the study this cohort. There were no birth defects reported in all the infants borne to these participants.
Adverse Reactions	2	1 severe GI symptoms (switched to TLE), 1 hepatoxicity (ART was temporality discontinued and participant was transferred to a higher-level facility where DTG was restarted)
Death	5	1 due to Immune Reconstitution inflammatory Syndrome (IRIS). 4 of the patients were ART naïve, 1 was ART experienced but had been on ART for less than 3 months.
Transferred	5	4 transferred to other clinics informally. 1 transferred formally
LTFU	23	Naïve = 17, Experienced = 6. Follow-up is in progress for the rest

### Results of patient interviews at month-one and month-six

A total of 319patients participated in the interviews at month-one and 297participated in the month-six follow-up interviews. Characteristics of patients who participated in the interviews are summarized in Table 3 below.

**Table 3:** Summary statistics for participants in interviews at month-one and month-six.

		Total	Treatment- Experienced	Treatment-Naive
**Month-one interviews**	Total	319	151 (47%)	168 (53%)
	Median Age (years)	35	36 IQR = 14	33 IQR 15
	Male	143	61 (43%)	88 (57%)
	Female	176	90 (51%)	90 (49%)
**Month-six interviews**	Total	297	150 (51%)	147 (49%)
	Median Age (years)	36	37 IQR = 16	33 IQR = 13
	Male	134	62 (46%)	72 (54%)
	Female	163	88 (54%)	75 (46%)

#### Patient acceptability

**Fig 1:**
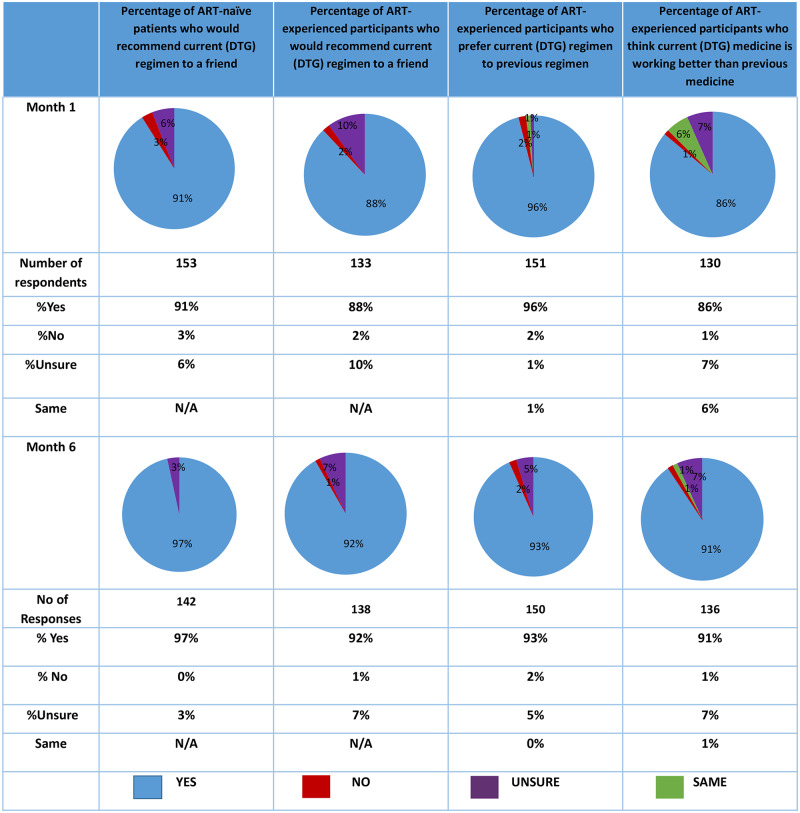
Responses to acceptability questions.

The results showed a high level of acceptability of a DTG-containing regimen for both treatment-naïve patients initiating on ART and treatment-experienced patients who switched from an NNRTI-based regimen at both one and six-months. See Fig 1 below.

#### Self-reported side effects

The rate of patients that self-reported at least one side effect was 43% and 33% at month-one and month-six, respectively. For both periods, the rates were higher for experienced patients than naïve patients. For both experienced and naïve patients, the proportion reporting any side effects and those who reported any severe side effects was less at 6 months compared to month one although the difference in reporting between both time periods was not significant. Side effects were considered severe if it was graded 4 or 5 out of 5. See Table 4 below:

**Table 4:** Patients reporting feeling any side effects or any severe side effects.

	Month-one interviews		Months-six interviews		
Patient Cohort	n	%	n	%	P-value
**Naïve**	**N = 168**		**N = 147**		
Any Side Effect	69	41%	43	29%	0.15
Any Severe Side Effect	17	10%	9	6%	0.28
**Experienced**	**N = 151**		**N = 150**		
Any Side Effect	69	41%	55	37%	0.22
Any Severe Side Effect	17	10%	11	7%	0.62
**All**	**N = 338**		**N = 297**		
Any Side Effect	137	43%	98	33%	0.06
Any Severe Side Effect	32	10%	20	7%	0.39

**Fig 2:**
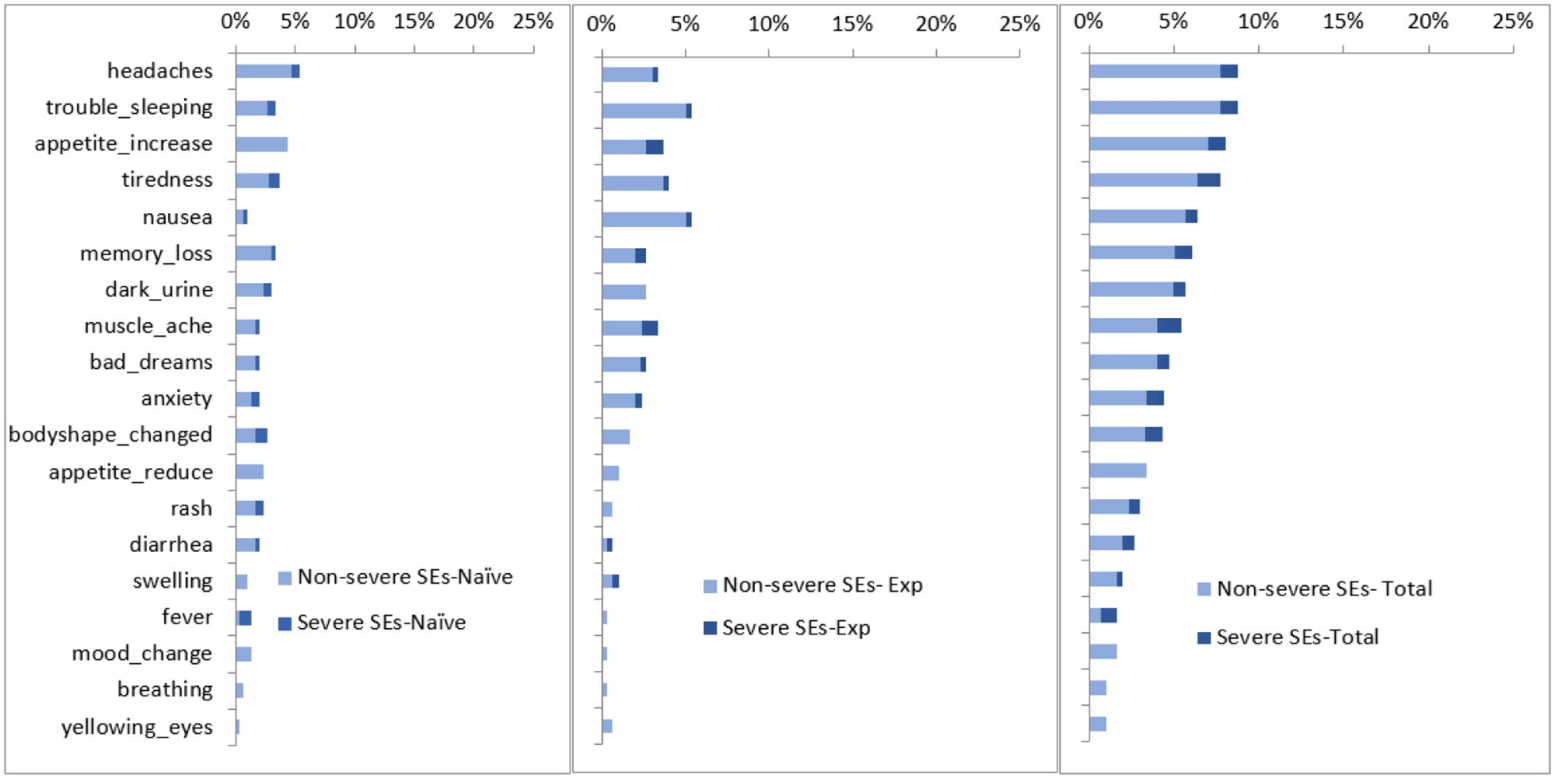
Reported side effects from most prevalent mentioned at month 6 for naïve patients (left) experienced patients (middle) and the total (right).

Fig 2 shows the reported side effects at month-six. Headaches (9%) was the most commonly mentioned side effect, followed by trouble sleeping. However, tiredness was the most commonly mentioned severe side effect. Increase in appetite, which was the most commonly mentioned side effect at the one-month interviews ranked third at month-six (8%).

The second question on side effects asked treatment-experienced ART patients to explain any changes in side effects they had experienced using their previous regimen since starting the new DTG-based regimen, and whether this pre-existing side effect had resolved, improved, remained unchanged or worsened. 533 side effects were reported by 116 patients at month-one while 287 side effects were reported by 79 patients at month-six. In both month-one and month-six interviews, more than 70% of side effects recalled by experienced patients were either improved or resolved while taking a DTG-based regimen. Table 5 summarizes the results.

**Table 5:** Progression of side effects reported by experienced patients.

	Month-one interviews		Month-six interviews	
	n	%	n	%
Number of side effects reported to be experienced on previous regimen	N = 518		N = 275	
Resolved	327	63%	166	60%
Improved	122	24%	72	26%
Did not change	34	7%	20	7%
Worsened	35	7%	17	6%

**Fig 3:**
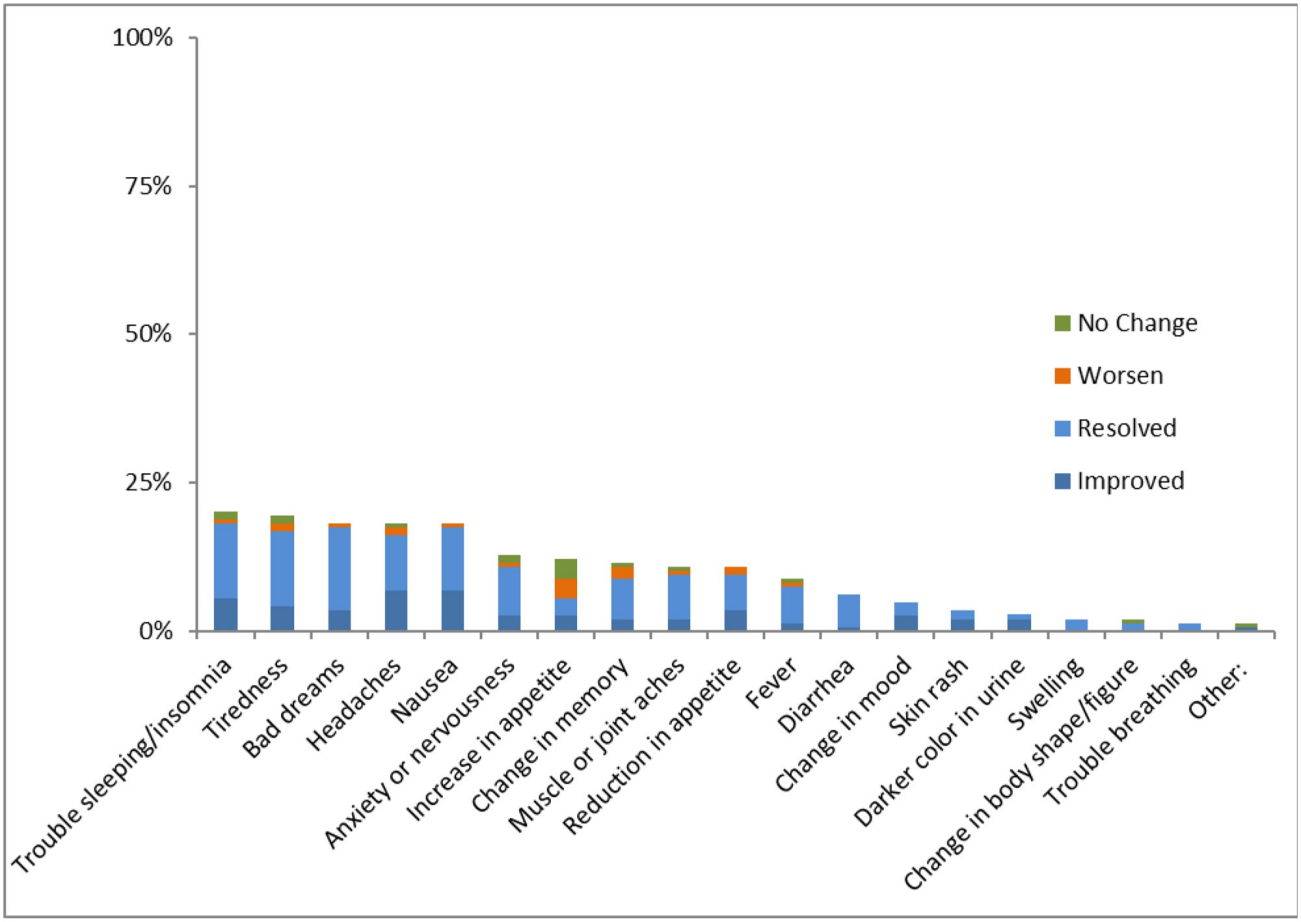
Side effects mentioned by experienced patients who report at least one side effect resolved, improved, did not change or worsened at 6 months.

There were differences amongst the side effects as shown by Fig 3 below. At 6-months, trouble sleeping was the most commonly mentioned previous side effect. Of this subset of respondents, 91% said the sleeping problems had either improved or resolved, and 6% said this had worsened since switching to DTG. The side effect that showed the least resolution was an increase in appetite; 12% of the respondents said they had this side effect on their previous regimen and 47% of these had improved or resolved, while 26% said that this had gotten worse.

#### Qualitative responses

The final question in the patients’ interviews asked the respondents an open-ended question to describe their thoughts on the regimen they are taking. The most common theme mentioned was that patients were experiencing fewer or no side effects and that the current regimen was preferred to the previous regimen–“*The medication has improved my general appearance I have not got any major side effects from it so I have no problem taking it*, *I take it easily*.” “*The medication has helped me a lot*. *The side effects I used to experience with the old medication have all resolved*. *The previous medication used to give me headache all the time*, *which is not the case with the current…”* Secondly many patients detailed positive experiences with regards to a smaller pill size, which made it easier to swallow. One patient remarked; “… *The current medicine is so small*. *It cannot be identified as ARV medication*. *It is so good*,*”* while another added that the additional pill burden of taking a dual and a single was not a problem: “*Am not worried of the pill burden anymore the size doesn’t worry me to swallow*.” Another patient pointed to the convenience of taking the drug, “…*……I used to miss (previous) doses because of getting home late*.*”*

There were a few patients that mentioned that they liked their new regimen despite the side effects, whilst some patients commented they liked the medication because the side effects from their previous regimen had either improved or resolved. One patient noted that, “*This new medicine may be working for me because my body is no longer itching too much*, *but the headache is still there*.” Another detailed, “It’s a good drug which is comfortable to swallow and has no side effects”. Some of the patients mentioned an increase in appetite as a positive side effect and associated it with an increase in energy. One said, “*This drug is really good because it has improved my appetite and body weight*.” While others associated this with feelings of hunger–“*Good medicine but (it makes me) feel a lot of hunger*.*”* Some patients expressed uncertainty about any unknown long term side effects: “*So far the drug is good and easy to take*. *My worry is about the long term side effects*.”

### Health record findings

#### Opportunistic infections

105 patients (29%) had at least one recorded opportunistic infection (OIs). Although statistically insignificant, slightly more OIs were recorded in the first visit (50%) compared to subsequent visits; 45% (p = 0.50), 43% (p = 0.89) at the second and third visits respectively. However,these were not necessarily the same patients or same OIs. Upper respiratory tract infections (URTIs) was the most frequently recorded OI; there were 34 patients who reported an URTI at least one visit, followed by tuberculosis (29 patients). Sixteen patients were diagnosed with a neuropsychiatric disorder. Other infections were reported by 29 patients during at least one of the visits many of the ‘other’ category includes oral candidiasis and oral thrush [7] and fevers [3].

#### Recorded side effects

**Fig 4:**
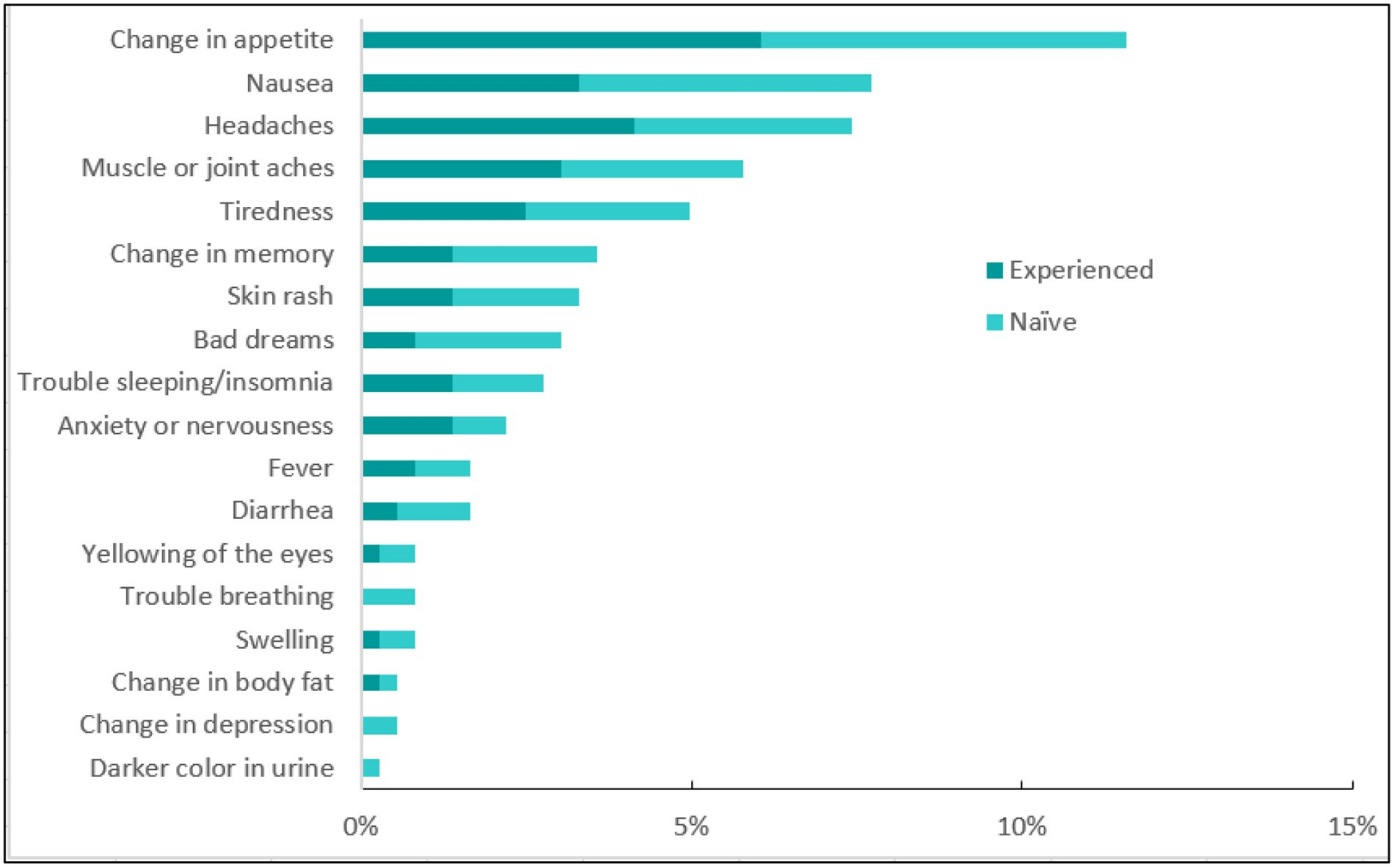
Side effects recorded in the patient records.

Any reported side effects were recorded in the patient’s clinical record at each clinic visit without regard to severity. We included each side effect that was mentioned on at least one visit, although some were mentioned more than once for a single patient. Overall 32% of the patients had at least one recorded side effect during at least one visit. Change in appetite was the most commonly recorded side effect in the patients’ health records at 12%, equally reported by treatment-experienced and naïve, followed by nausea and headaches, 8% and 7%, respectively. Other recorded side effects include joint and muscle aches (6%), tiredness and depression recorded in 6%, 5% and 4% of the patient records, respectively. Insomnia was recorded in 3% of patient records during at least one visit. See Fig 4.

#### Virological outcomes

There were 285 records that included viral load results at 6 months. Nearly all patients were virally suppressed at 6 months (94%) defined as 1000 copies/ mL. Six treatment-experienced patients (4%) had a viral load over 1000 copies/mL compared to 11 (7%) of the treatment naïve patients with viral load results (See Table 6).

**Table 6:** Viral suppression results by ART status at enrolment.

Patient Cohort	<1000 copies/mL		Over 1000 copies/mL	
	n	%	n	%
Treatment-Experience	132	96%	6	4%
Treatment-Naïve	136	93%	11	7%
Total	268	94%	17	6%

Three of the unsuppressed subset were reported to have had TB in their health records and 3 others were reported to have had depression during at least one visit in the follow-up period. Health record forms also reported side effects including fever (two patients) and nervousness (two patients) amongst unsuppressed patients during the period of follow-up.

16 of the 17 patients with unsuppressed viral loads participated in the acceptability interviews at 6 months. Eight of the patients self-reported to have at least one side effect and one reported at least one severe side effect (bad dreams and trouble sleeping/insomnia). All of the patients reported that they would recommend the drug to a friend. Although adherence is likely to be suboptimal amongst these patients, only two reported that they had missed a dose in the last seven days. The qualitative responses in the interviews did not point to negative experiences with the regimen; all reported no complaints and/or a positive experience.

Refer to the zip file labelled “S1 data” for the data from which results above have been derived and the file labelled “S2 Data” for the accompanying codebook.

## Discussion and conclusions

### Discussion

Overall, the study findings show a very high level of acceptability of DTG-based regimens across both experienced and naïve patients. This may be as a result of the reduced number of side effects, the smaller pill size, or because their experience with DTG was more favorable than their previous regimen. It is noted however that the for the treatment-experienced sub-group, the study enrolled only those patients who were already experiencing side effects on their previous regimen and as a result these patients may be more likely to show bias favoring DTG compared to treatment naïve patients.

Across both patient groups and both surveys, at least one side effect was reported by at least a third of the patients interviewed. The frequencies at month-six were lower than the frequencies reported at month-one although the difference was not statistically significant.

Alternatively, since patients experiencing side effects were preferentially recruited for this study, the participants may represent a cohort more likely to experience or report side effects. Additionally, adverse events tend to occur in the early stages of a new medication and improve over time. In the month-six survey 84% of treatment-experienced patients indicated side effects were either improving or resolved, likely contributing to the high levels of patient acceptability seen in the study.

During the 6 months interviews, the most commonly mentioned side effect was trouble sleeping, which is in line with literature published from other findings [14]. Although this is the most frequently mentioned side effect and amongst treatment-experienced patients, it should be noted that most of those patients had experienced trouble sleeping from their previous regimen and reported it to be improved or resolved with DTG. This suggests that that DTG can improve insomnia compared to EFV as 93% over the experienced patients switched to DTG from an EFV based regimen.

The other commonly mentioned side effects was “increase in appetite”, which had not been mentioned in studies on DTG published before the start of the pilot. This study, did not assess the relationship between increase in appetite and increase in body weight. However Norwood J et al published a study suggesting an increase in body weight among patient using DTG-based regimen [15]. Another cohort study also reported an increase in weight associated with DTG-based regimen especially amongst women and in patients taking DTG with Abacavir [16]. Data from the SCOLTA cohort revealed significant increases in body mass index (BMI) within one year in patients treated with DTG (P = 0.004), which correlated with low baseline BMI and older age [17]. Recently trials, ADVANCE and NAMSAL, conducted in South Africa and Cameroon respectively reported higher weight gain in the TDF/FTC/DTG arm compared to patients in the TDF/FTC/EFV arm [18]. The long term effects of the increased appetite reported in our study and weight gain reported in other studies require further evaluation.

We found that 94% of the patients achieved viral suppression, which is higher than the estimated national average of 88% [19]. This is consistent with previous studies which have shown DTG-based regimen to achieve high viral suppression rates [14, 20]. Since the study follow-up period was only 6 months, the long-term effect of DTG-based regimen on viral suppression was not assessed.

#### Limitations

There were a number of limitations with the study, the first is that due to resource constraints and timing we decided that health care workers were the best positioned to administer the patient acceptability questionnaire, which could result in some response bias from patients. The interviews were designed for quantitative trend analysis and hence consisted of multiple answer questions that may have missed some important opinions. For example, the side effects noted were already listed for patients; although there was an ‘other’ option, it was not always utilized. Secondary, for the ART experienced group, the study enrolled only those patients who were already experiencing side effects on their previous regimen which made them more likely to show bias favoring DTG. Thirdly, although we observed some trends suggesting that side effects’ reporting reduced between two and six months, our study was not powered to look at differences in side effects’ reporting overtime. This is an area that studies should explore further especially for side effects like “increase in appetite” that could significantly affect long-term health outcomes. Lastly, since the study follow-up period was only 6 months, the long-term effect of DTG-based regimen on viral suppression was not assessed.

### Conclusions

Overall, there was a high acceptability of DTG-based regimen amongst patients despite the relatively high rate of side effects reported. Further evaluations are needed to assess the reported ‘increased appetite’ and its short and long term effects on weight or body mass index. The majority of patients in this cohort achieved viral suppression at 6 months.

## Supporting information

S1 Data (ZIP)

S2 Data (ZIP)
